# A Composite Pulse Excitation Technique for Air-Coupled Ultrasonic Detection of Defects in Wood

**DOI:** 10.3390/s24237550

**Published:** 2024-11-26

**Authors:** Jun Wang, Changsen Zhang, Maocheng Zhao, Hongyan Zou, Liang Qi, Zheng Wang

**Affiliations:** 1 College of Information Science and Technology, Nanjing Forestry University, Nanjing 210037, China; wangjun@njfu.edu.cn (J.W.); zhangcs@njfu.edu.cn (C.Z.); 2 College of Mechanical and Electronic Engineering, Nanjing Forestry University, Nanjing 210037, China; zouhy@njfu.edu.cn (H.Z.); liangqi@njfu.edu.cn (L.Q.); 3 College of Materials Science and Engineering, Nanjing Forestry University, Nanjing 210037, China; wangzheng63258@163.com

**Keywords:** composite pulse excitation, air-coupled ultrasound, nondestructive testing

## Abstract

To overcome the problems of the low signal-to-noise ratio and poor performance of wood ultrasonic images caused by ring-down vibrations during the ultrasonic quality detection of wood, a composite pulse excitation technique using a wood air-coupled ultrasonic detection system is proposed. Through a mathematical analysis of the output of the ultrasonic transducer, the conditions necessary for implementing composite pulse excitation were analyzed and established, and its feasibility was verified through COMSOL simulations. Firstly, wood samples with knot and pit defects were used as experimental samples. We refined the parameters for the composite pulse excitation technique by conducting A-scan measurements on both defective and non-defective areas of the samples. Moreover, two stepper motors were employed to control the path for C-scan imaging to detect wood defects. The experiment results showed that the composite pulse excitation technique significantly enhanced the precision of nondestructive ultrasonic testing for wood defects compared to the traditional single-pulse excitation method. This technique successfully achieved precise detection and location of pit defects, with a detection accuracy rate of 90% for knot defects.

## 1. Introduction

As wood grows, environmental factors and biological damage frequently result in defects such as knots and pits [1,2,3]. To improve wood utilization and reduce costs, ultrasound testing is widely employed in wood quality inspection due to its high energy, strong penetration ability and capacity to effectively reflect defect information within the wood [4,5,6]. Currently, ultrasound defect detection in wood primarily relies on energy attenuation or velocity changes to indirectly assess wood quality [7,8,9,10]. Traditional ultrasound testing methods include contact and immersion techniques, both of which require the use of a couplant or ultrasound transmission on the wood surface [11,12]. However, these liquid couplants have the potential to contaminate the wood. By comparison, air-coupled ultrasound inspection technology enables nondestructive testing by eliminating contact between the probe and the sample and it has been successfully applied in food and composite material testing [13,14,15,16]. In wood ultrasound inspection, however, the superposition of the ring-down vibration signal with the effective detection signal makes the signal analysis complicated and it is difficult to distinguish between defect signals. Furthermore, the ring-down vibration signal may generate additional noise and artifacts that impair imaging accuracy. The term “ring-down vibration” usually refers to the continuous vibration phenomenon produced by an ultrasonic transducer due to its inherent vibration modes after the excitation signal from the transducer is stopped. This vibration is known as the transducer’s ring-down vibration. Therefore, suppressing the ring-down vibration of the ultrasound transducer is essential for improving detection accuracy.

Various methods have been introduced to reduce the effects of ring-down vibration. Kusano et al. proposed improving the oscillation time of Piezoelectric Micromachined Ultrasonic Transducers (PMUTs) by adjusting the polarization direction of the piezoelectric material [17,18]. Li et al. subsequently accelerated the attenuation of the ring-down vibration signal by increasing the attenuation coefficient of the circuit loop [19]. Other methods, such as VSC control [20] and broadband attenuation strategies [21], have also been employed to enhance oscillation decay performance. Chang et al. proposed using parallel resistors to reduce the DC time constant of transient responses [22,23]. Yao et al. developed a signal compensation technique based on time-varying gain theory that can automatically adjust the gain to reduce blind spots [24]. Wu et al. proposed a decay suppression system based on transfer functions, reducing the oscillation time of a 115 kHz PMUT array by up to 93% [25]. Yang et al. applied resonant noise-matching techniques using a resonant LC network to increase the input impedance of the sensor and reduce ringing in the signal, thereby improving the image resolution [26]. While these methods enhance the ultrasound transducer performance, they also increase the system complexity and cost by adding external circuitry. Some studies suggest that residual oscillations can be reduced by adjusting the transmit power and gain, optimizing the coding, and actively attenuating reception, but it remains challenging to accurately control oscillation cessation and the configuration process is complex [27,28,29,30,31,32]. The research has indicated that employing double-pulse transmission in short coded sequences significantly reduces noise levels and yields higher signal-to-noise ratios compared to single-pulse transmission for Golay codes of equivalent length [33]. This provides an effective reference for applying composite pulse excitation technology to wood air-coupled nondestructive testing, addressing the issues of low signal-to-noise ratios and poor defect detection performance.

In order to suppress the effect of ultrasound transducer ring-down vibration and improve the accuracy of ultrasound nondestructive detection of wood defects, this paper discusses a composite pulse excitation technique applied to the air-coupled ultrasound detection of wood. Using composite pulse excitation of a low-frequency ultrasound transducer, a performance similar to that of a high-frequency ultrasound transducer can be achieved while maintaining a low energy attenuation and excitation voltage. This method simplifies the system structure without compromising the transducer’s performance, thereby improving the accuracy and reliability of ultrasound detection. Compared to single-pulse excitation, composite pulse excitation technology significantly enhances the accuracy of ultrasound nondestructive testing of wood defects, achieving a detection accuracy of up to 90% for knots and enabling the precise detection and localization of pit defects.

## 2. Principle of Air-Coupled Ultrasound Detection with Composite Pulse Excitation

### 2.1. Composition of Air-Coupled Ultrasound Detection System with Composite Pulse Excitation

**Figure 1:**
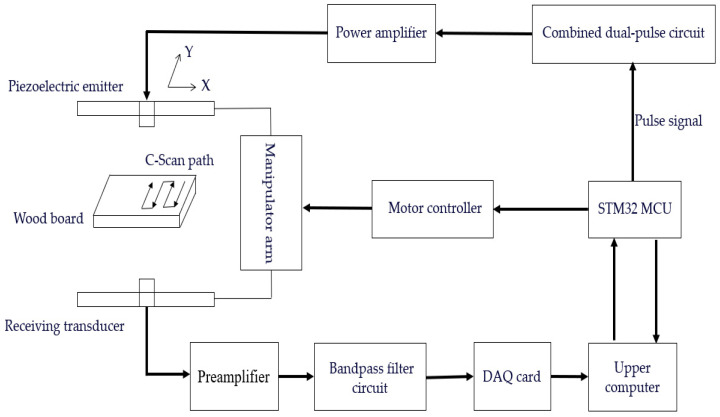
Flowchart of air-coupled ultrasound inspection system with composite pulse excitation.

In this study, an air-coupled ultrasonic wood detection system with composite pulse excitation was designed, as shown in Figure 1. The system consists of a main controller and a microcontroller, which is responsible for generating the required pulse signals. The amplitude ratio of the two pulse signals can be precisely adjusted using a resistive voltage divider circuit to produce a composite pulse excitation signal suitable for the ultrasound transducer. A power amplifier circuit amplifies the composite pulse signals, increases the gain, and raises the voltage to approximately 150 V to meet the excitation requirements of the ultrasound transducer for penetrating the wood. When the transducer is excited, the piezoelectric film within it generates ultrasound waves through a combination of elastic and resistive forces [34]. Due to the acoustic impedance mismatch between wood and air, the ultrasound signals are significantly attenuated during propagation, resulting in weak transmitted signals accompanied by substantial noise. To effectively extract and process the received ultrasound signals, the system employs a preamplifier. This preamplifier not only provides preliminary DC isolation filtering of the received signal, but it also significantly enhances the signal amplitude. Additionally, an active bandpass filter circuit is employed to filter out both high- and low-frequency noise. The OSCHO2 data acquisition card is responsible for acquiring and converting the amplified and conditioned signals and transmitting the data to the host computer. The host computer then sends the target scanning position command to the microcontroller to control the motor’s running speed, thereby scanning each sample point on the wood. After scanning, the host computer performs a correlation analysis on the collected data and uses the maximum value of the correlation result as the image data for the sampling points, enabling the real-time imaging and visualization of wood defects.

**Figure 2:**
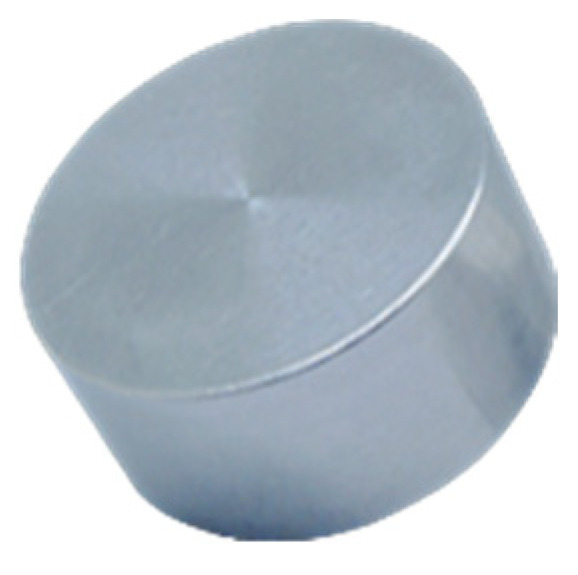
Picture of ultrasonic transducer.

A piezoelectric ultrasound transducer, model 40A25TR-1 from Hengchuang Sensing Co., Ltd. (Shenzhen, China), was employed in this system, as illustrated in Figure 2. It has a probe diameter of 25 mm and a thickness of 12 mm. This ultrasonic transducer provides up to −74 dB sensitivity at an operating frequency of 40 kHz, offering excellent narrowband responses and strong energy directivity. However, when ultrasound propagates through wood, the received signal contains not only the target ultrasound signal but also significant high-frequency and low-frequency noise components, along with a DC-biased signal, originating from the ultrasound probe’s properties and possible scattering effects during propagation. To effectively extract the target signal and eliminate noise, this system incorporates a preamplifier to perform isolation and further amplify the signal. The AD8421 amplifier, a high-performance instrumentation amplifier from Analog Devices (Wilmington, MA, USA), features extremely low input noise, a low bias current, and excellent common-mode rejection with gains up to 10,000×, making it ideal for processing weak signals. This amplifier provides high-precision amplification of ultrasound signals by effectively suppressing both high- and low-frequency noise, ensuring signal clarity and stability. After amplification, low- and high-frequency noise components remain in the output signal, which degrade the system’s signal-to-noise ratio. To further filter unwanted frequency components, the LT1567 amplifier from Analog Devices (Milpitas, CA, USA) is employed as the bandpass filter in this design. The LT1567 amplifier is a low-noise, high-speed, broadband operational amplifier that facilitates the construction of flexible second-order bandpass filters through the careful selection of external resistors and capacitors. The bandpass filter was designed with a center frequency of 40 kHz, a −3 dB bandwidth of 16 kHz, and a quality factor (Q) of approximately 2.5. The filter effectively passes signals within the 40 kHz band while suppressing interfering noise in other bands, thereby ensuring effective transmission and signal quality of the output from the receiver transducer. By combining signal isolation, amplification, and band-pass filtering, this system significantly improves the quality of ultrasonic signals, optimizes the signal-to-noise ratio, and ensures high efficiency and stability in weak signal processing.

### 2.2. Introduction to Composite Pulse Excitation Technology

**Figure 3:**
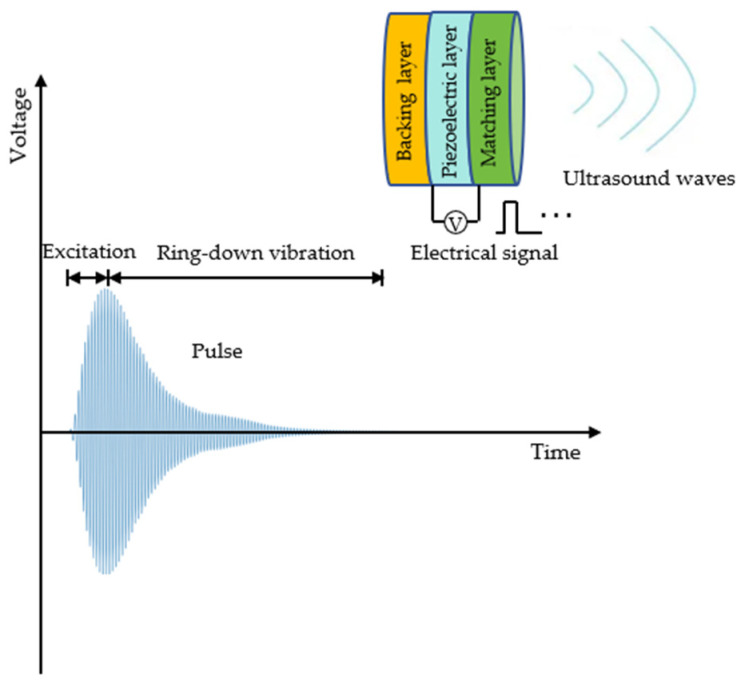
Schematic diagram of ring-down vibration trailing.

In ultrasound inspections, signals received from a low-frequency ultrasound transducer typically exhibit significant ring-down vibration effects (see Figure 3). This ring-down effect diminishes the prominence of the signal’s main peak, thereby reducing the axial resolution of the ultrasound transducer. The ring-down vibration fails to accurately represent the propagation time and signal amplitude, complicating signal processing. In contrast, the ring-down time of the received signal from a high-frequency ultrasound transducer is shorter, with a more pronounced main peak and higher axial resolution. However, high-frequency ultrasound waves experience greater energy attenuation and have poorer penetration in wood, resulting in weaker received signals. This necessitates that the detection circuit has increased sensitivity and accuracy to effectively capture these weak signals. To optimize signal quality and enhance axial resolution, this paper proposes a composite pulse excitation technique. The technique aims to retain the portion of the received signal from the start to the peak while effectively suppressing the residual ring-down vibration after the peak. By combining dual-pulse excitation with low-frequency ultrasound transducers, a performance comparable to high-frequency transducers can be achieved, while maintaining a lower energy attenuation and excitation voltage. This approach not only simplifies the system structure but also improves the accuracy and reliability of ultrasound detection.

**Figure 4:**
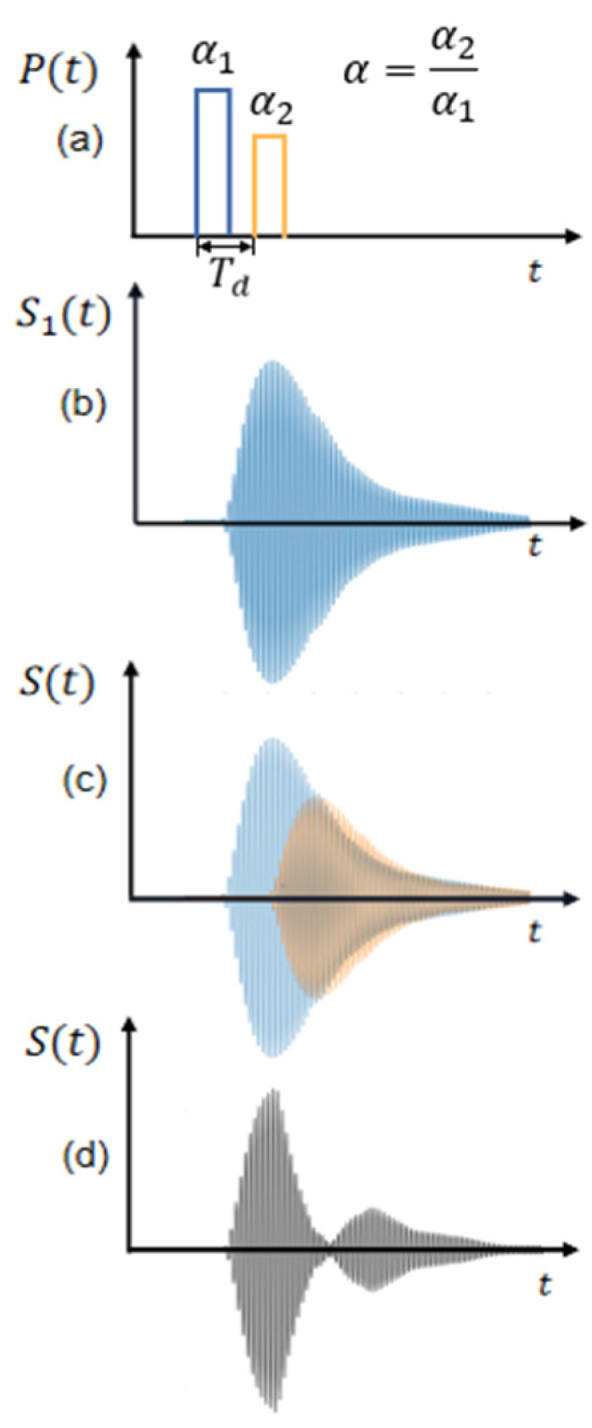
Composite pulse excitation schematic diagram: (**a**) Composite pulse signal used to excite the transducer, (**b**) Output signal of the sensor after the first pulse excitation, (**c**) Each of the two pulses excites the output signal of the transducer, and (**d**) The output signal of the ultrasonic transducer excited by the composite pulse.

In this paper, a composite pulse excitation technique is proposed. The composite pulse excitation technique primarily involves combining two pulses. Both pulses have equal widths, with the amplitude of the second pulse being *α* times that of the first pulse, and delayed by $T_{d}$ seconds relative to the first pulse. When these combined pulses are applied to the piezoelectric ultrasound transducer, the corresponding output S(t) is obtained, as shown in Figure 4c. Compared with the first pulse output $s_{1}\left(t\right)$ of the single-excitation ultrasonic transducer, the ring-down vibration signal following the peak of the signal received by the composite pulse excitation transducer is significantly suppressed, as shown in Figure 4d.

Since the pulse width of the second pulse is the same as that of the first pulse, but its amplitude is α times that of the first pulse, the received waveform under the excitation of the second pulse can be regarded as a scaled-down version of the received waveform from the first pulse excitation. The second pulse is delayed by $T_{d}$ seconds relative to the first pulse, and its received waveform is correspondingly delayed. Based on the principle of linear superposition of acoustic waves, the response of the ultrasound transducer can be precisely controlled by adjusting the amplitude and delay time of the pulse to meet specific waveform requirements.

To eliminate the ring-down signal after the highest peak of the received waveform from the first pulse, it is essential to precisely adjust the delay time between the first pulse and second pulse, as well as control the peak amplitude of the second pulse. When the delay time *T_d_* of the second pulse is equal to the time interval from the starting point to the highest peak in the first pulse’s received waveform, the highest peak of the second pulse is delayed by time *T_d_* relative to the highest peak of the first pulse.

**Figure 5:**
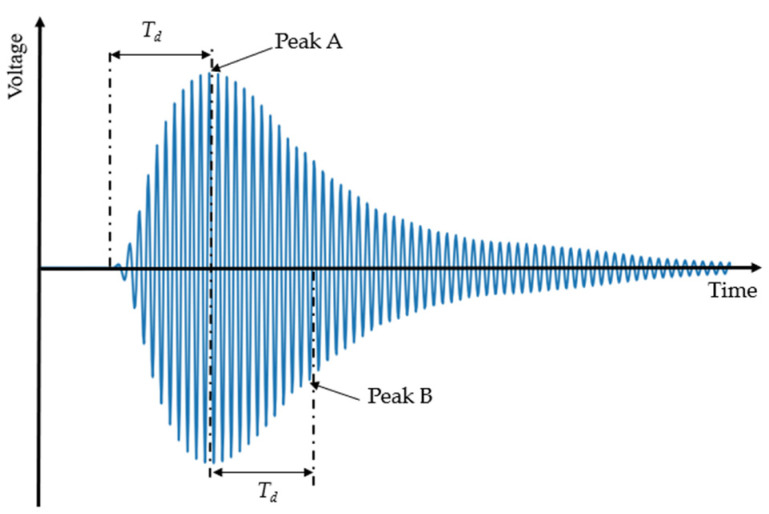
Single-pulse excitation of ultrasound transducer.

In order to achieve complete elimination of the signal at point B in the first pulse’s received waveform, the peak amplitude of the second pulse should be set to the absolute value of peak B in the first pulse’s received waveform. This will determine the value of α. When the first pulse excites the ultrasound transducer and there are *x* peaks before the maximum positive peak in the received waveform, then $T_{d}$ is equal to *xT + T/2*. The peak value of peak *A* is $A_{1}$, and the peak value of peak *B* is $A_{2}$, so that α = $A_{2}$/$A_{1}$, as shown in Figure 5. The α and *n* values determined are used as parameters of the second pulse, and the composite pulse excites the ultrasound transducer to achieve suppression of the ringing vibration.

### 2.3. Theoretical Analysis

To investigate the suppression of ring-down vibration effects in ultrasonic transducers, the output of the transducer is analyzed using mathematical methods. Let s(t) represent the input of the first pulse, and h(t) represent the impulse response of the transducer, while y(t) denotes the output of the transducer when the input consists of two pulses. Since the second pulse has the same duration as the first pulse, with an amplitude α times that of the first pulse and delayed by $T_{d}$ seconds, the output y(t) can be expressed as

(1) $$y(t)=[s(t)+\alpha \cdot s(t-T_{d})]*h(t)$$

In the frequency domain, let *k*(*t*) = $s(t-T_{d})$. The Fourier transforms of *k*(*t*) and y(t) are given by

(2) $$K(\omega )=e^{-jwT_{d}}S(\omega )$$

(3) $$Y(\omega )=S(\omega )\cdot [1+\alpha \cdot e^{-jwT_{d}}]\cdot H(\omega )$$

From Equation (3), it can be seen that the parameters α and $T_{d}$ affect the transducer output in the frequency domain. Let

(4) $$G(\omega )=1+\alpha \cdot e^{-jwT_{d}}$$

Substitute in Euler’s formula ($e^{-j\theta }=cos\theta -jsin\theta$):

(5) $$G(\omega )=1+\alpha (cos(\omega T_{d})-jsin(\omega T_{d}))$$

Separate into real and imaginary parts:

(6) $$G(\omega )=(1+\alpha cos(\omega T_{d}))-j\dot{\alpha }sin(\omega T_{d})$$

(7) $$|G(\omega )|=\sqrt{{\left[1+\alpha cos(\omega T_{d})\right]}^{2}+{\left[\alpha sin(\omega T_{d})\right]}^{2}}$$

Since ω=2πf, substitute $\omega T_{d}$ with $2\pi fT_{d}$:

(8) $$|G(f)|=\sqrt{{\left[1+\alpha cos(2\pi fT_{d})\right]}^{2}+{\left[\alpha sin(2\pi fT_{d})\right]}^{2}}$$

(9) $$=\sqrt{1+2\alpha cos(2\pi fT_{d})+\alpha^{2}{cos}^{2}(2\pi fT_{d})+\alpha^{2}{sin}^{2}(2\pi fT_{d})}$$

From the trigonometric constant ${cos}^{2}(x)+{sin}^{2}(x)=1$, then

(10) $$|G(f)|=\sqrt{1+\alpha^{2}+2\alpha \cdot cos(2\pi fT_{d})}$$

Since cos(*n*π) = ±1, $f=n/2T_{d}$. When 0 < *α* < 1, *n* is a positive integer.

When *n* is an even value, *n* = 2X and X is any positive integer:

(11) $$|G(f){|}_{max}=1+\alpha ,f=X/T_{d},\;T_{d}=\frac{n}{2}T=XT$$

When *n* is an odd value, *n* = 2X+1 and X is any positive integer:

(12) $${\left|G(f)\right|}_{min}=1-\alpha ,f=(2X+1)/2T_{d},\;T_{d}=\frac{n}{2}T=\frac{2X+1}{2}T$$

Based on the derived Formulas (11) and (12), the maximum and minimum values of the transducer’s output depend on the parameter α, while $T_{d}$ only determines the frequency at which these extrema occur. To suppress the pulse, the value of |G(f)| needs to be minimized. When 0 < α < 1 and n is odd, the value of |G(f)| is less than 1, resulting in a weaker frequency response, which effectively reduces the ringing effect and shortens the signal duration.

## 3. Simulation Test

**Figure 6:**
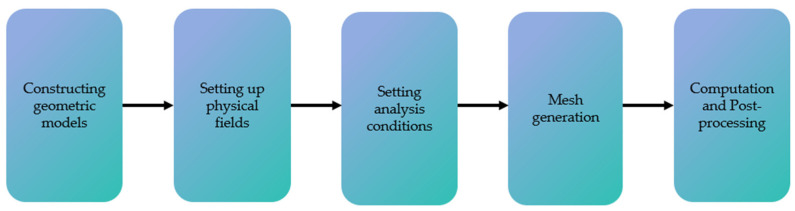
COMSOL modeling simulation flow.

By creating a finite element model in COMSOL 6.1 software, different physical fields can be coupled to simulate and analyze complex environments. The modeling process, shown in Figure 6, includes the following steps: constructing the geometric model, defining the physical fields, setting the analysis conditions, meshing, configuring the solver, and post-processing.

**Figure 7:**
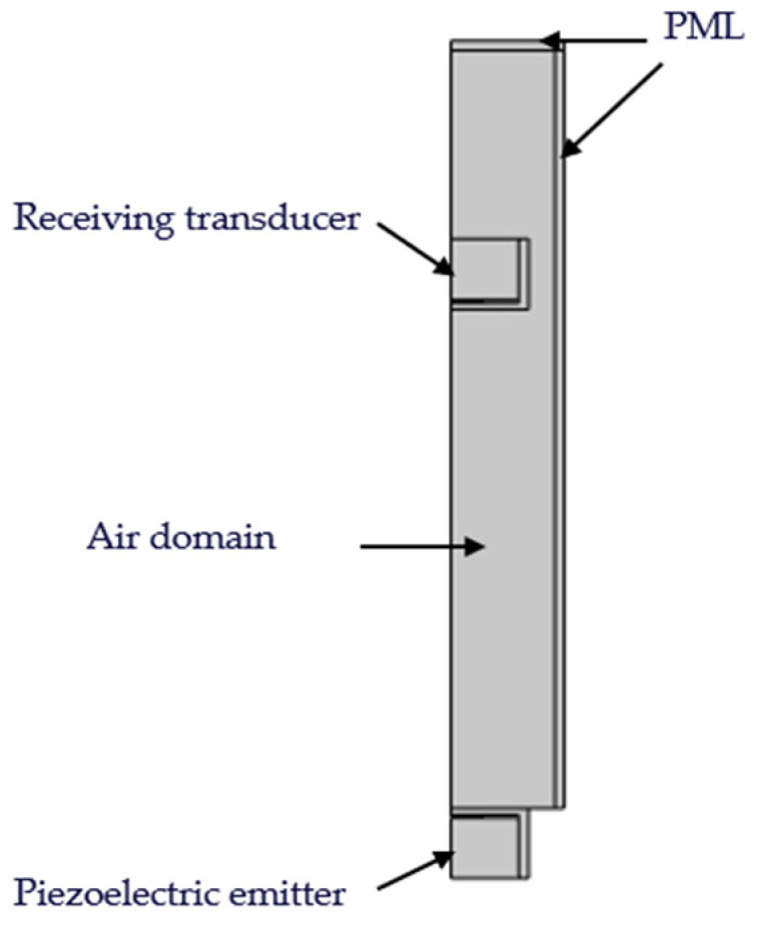
Simulation model diagram.

In this study, a two-dimensional axisymmetric model was employed to simulate the piezoelectric transducer. The established simulation model is shown in Figure 7. In the model, the distance between the two transducers is 8 cm, and the dimensions of the air domain are 17.5 mm × 110 mm. Because the ultrasound waves generated by the transmitting transducer propagate in a finite domain, waves reflected from the boundaries can interfere with the signals at the receiving end. To reduce this interference, a Perfectly Matched Layer (PML) was implemented on the upper and right boundaries of the air domain. The PML serves as an approximate ideal absorber or radiator, simulating attenuation and absorption as the waves propagate towards infinity. This ensures that waves entering the PML gradually attenuate and become fully absorbed, preventing reflections back into the studied region and thereby minimizing the impact of boundary reflections on the received signals.

After the model is constructed, the physical fields of each module are set up, and two modules (pressure acoustics and solid mechanics) are used in this model. Specifically, the material properties of the two ultrasound transducers are incorporated into the solid mechanics module, where the PZT material inside the ultrasound transducer is modeled as a piezoelectric material and an electrostatic portion. The transmitting transducer uses the inverse piezoelectric effect to convert electrical energy into mechanical energy (ultrasound waves). Therefore, “ground” and “electric potential” boundaries are set on either side of the transducer. The receiver transducer converts acoustic waves into electrical energy using the positive piezoelectric effect, with a “ground” boundary and a “charge” boundary on each side. The air domain belongs to the Pressure Acoustics module, and the contact boundary between the air domain and the ultrasound transducer is set as an acoustic–structural boundary to couple these two physical fields. This model setup aims to accurately simulate the propagation behavior of ultrasound waves in practical applications and effectively reduce interference signals caused by boundary reflections, thereby enhancing the accuracy of the simulation results.

In the study of ultrasound propagation behavior in the air, the mesh size is critical to ensuring accurate calculation results. If the mesh size is too large, the calculation results may become imprecise or result in errors. Conversely, if the mesh size is too small, it will result in excessively long calculation times and consume excessive computational resources. It is generally required that the mesh size be one-tenth of the minimum wavelength in the excited mode during meshing. Therefore,

(13) $$L\leq \frac{\lambda_{min}}{10}=\frac{8.5\;mm}{10}=0.85\;mm$$

In the simulation model, the criterion for selecting the solution time step is that it must be shorter than the time it takes for the fastest wave to propagate through the smallest grid cell length. Therefore, to improve computational speed, the time step should be carefully selected to meet this requirement:

(14) $$\Delta t\leq \frac{L_{min}}{c_{max}}=\frac{0.85\;mm}{340\;m/s}=2.5\;\mu s$$

In this study, the time step of the solver was set to 1.25 μs to meet the experimental requirements.

**Figure 8:**
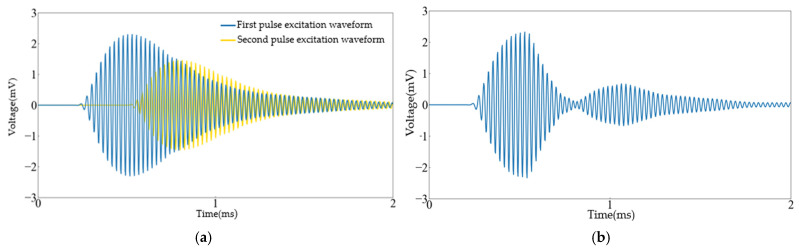
Simulation results graph: (**a**) Received waveform of an ultrasound transducer excited by two pulses separately and (**b**) received waveform of an ultrasound transducer excited by a composite pulse.

In the model, the terminal voltage of the transmitting transducer was set to 10 V with a pulse width of 12.5 μs. To optimize signal quality and eliminate the signal portion following the highest peak generated by the transducer after the first pulse excitation, the signal waveform data of the first pulse were captured and saved using MATLAB 2022 software. Based on the principle of composite pulse excitation, a second pulse was designed with its amplitude set to 6.4 V, the pulse width the same as the first pulse, and it lags behind the first pulse by 10.5 cycles. By using composite pulse signals to excite the transmitting transducer, the waveform of the receiving transducer, as shown in Figure 8b, significantly suppresses the ring-down vibration after the highest peak, thereby improving the signal quality.

To verify the effects of the delay time $T_{d}$ and the amplitude ratio α on the experimental elimination results, several sets of simulation experiments are performed using COMSOL software to evaluate the influence of $T_{d}$ and α on the ringing vibrations.

**Figure 9:**
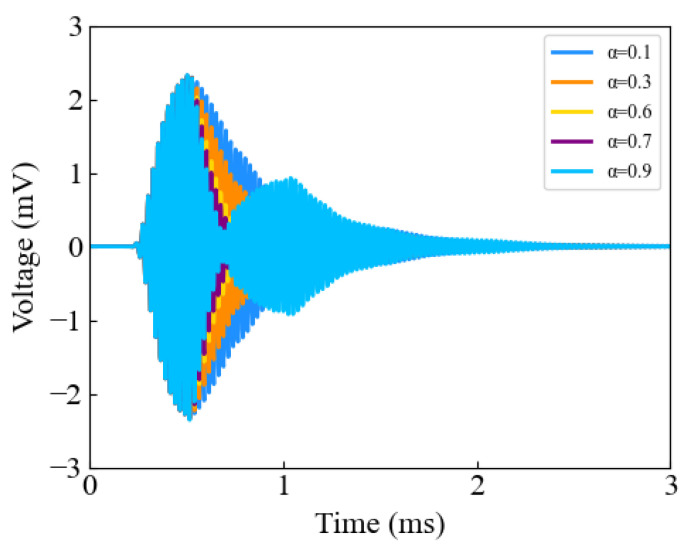
The effect of parameter α on inhibiting ringing vibration.

First, the amplitude ratio α between the second pulse and the first pulse amplitudes was adjusted using the first pulse excitation waveform in Figure 8a as a reference, with the delay time held constant. As shown in Figure 9, when the amplitude of the second pulse was relatively small compared to that of the first pulse, the attenuation effect on the trailing portion of the ring-down vibration in the ultrasound received signal was negligible. Conversely, when the amplitude of the second pulse was significant, although the attenuation effect improved, there may be an increase in the trailing waveform of the ring-down vibration. Therefore, when selecting the value of α, it is essential to first avoid an increase in residual vibration trailing caused by excessive amplitude, and then optimize α to improve the attenuation of ringing vibrations as much as possible.

**Figure 10:**
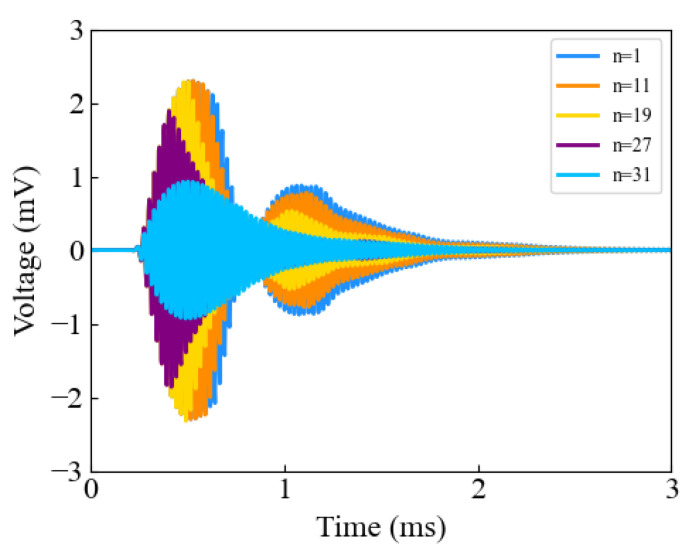
The effect of parameter n on inhibiting ringing vibration.

Secondly, the effect of delay time on the ring-down vibrations of the transducer was verified by keeping α at 0.6 and varying the value of the integer n. As shown in Figure 10, when the delay of the second pulse was small compared to the first pulse, the highest peak in the ultrasound received signal was eliminated; when the delay was large, the suppression effect on the ring-down vibrations was less pronounced. The value of n should first ensure that the highest peak in the ultrasonic received signal is not eliminated, and then aim to suppress the residual ring-down vibrations.

This verification process demonstrates the efficacy of composite pulse excitation technology and establishes a foundation for the further optimization of signal quality. Based on practical application requirements, the parameters of the pulse signal, such as the amplitude ratio and delay time, should be further optimized to maximize suppression performance.

## 4. Experimentation and Analysis

### 4.1. Experimental Setup

**Figure 11:**
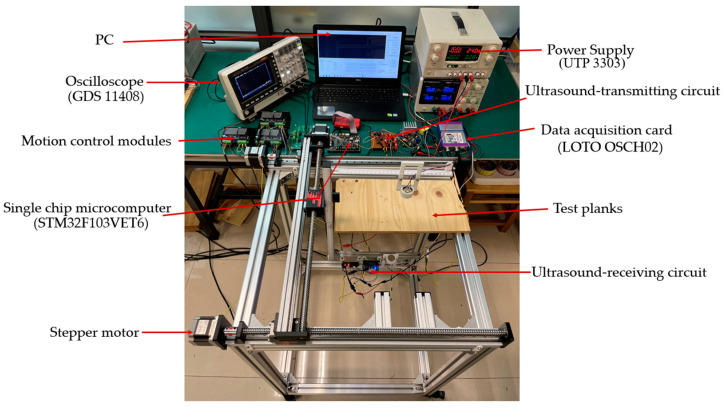
Full view of the detection system.

To verify the practical application of the composite pulse excitation technique in air-coupled ultrasound inspection, two distinct cedar boards were chosen as inspection specimens for this study, with the inspection area dimensions for both specimens established at 5 cm × 5 cm × 1 cm. In the experiment, the distance between the transducers of the transmitter and receiver and the wood panel sample significantly affected the detection results, including signal strength, noise interference, resolution, and imaging quality. Therefore, the appropriate adjustment of this distance is essential to ensuring signal quality and detection accuracy. In this study, the distance from the transmitting transducer to the sample was maintained at 6 cm, while the distance from the receiving transducer to the sample was maintained at 8 cm. The configuration of the entire detection system is illustrated in Figure 11.

### 4.2. Selection of Signaling Parameters

**Figure 12:**
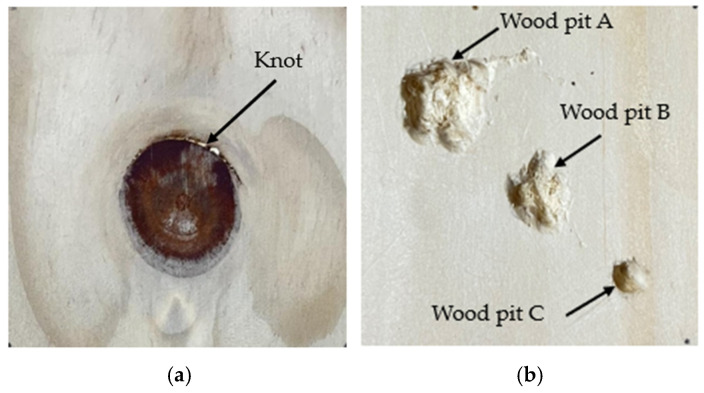
Diagram of physical wood defects: (**a**) Wood with knot defects and (**b**) wood with pit defects.

To determine the parameters of the composite pulse excitation signal for the test samples, ten preliminary test points were selected on two wood samples, covering knots, pits, and defect-free regions. Two wood samples are shown in Figure 12. A-scans were performed using a single pulse as the excitation signal, and the parameters of the double-pulse excitation for each point were determined based on the ultrasound signals received from the ten preliminary test points. The signal parameters were selected according to the principle of using the maximum delay and the minimum amplitude ratio to avoid the loss of the signal’s highest peak and the amplification of trailing effects.

Therefore, the first to fifth points in Table 1 are the detection points for defective wood knots, and the sixth to tenth points are the detection points for non-defective wood. According to Table 1, the parameters of the composite pulse excitation signal for the samples containing defective wood knots were determined as follows: the delay time $T_{d}$ was set to 180 μs and the amplitude ratio α was set to 0.6.

The first point to the third point in Table 2 were the detection points for wood pit A, the fourth point to the fifth point were the detection points for wood pit B, the sixth point was the detection point for wood pit C, and the seventh point to the tenth point were the detection points for wood without defects. According to Table 2, the parameters of the composite pulse excitation signals for the samples containing pit defects were as follows: the delay time $T_{d}$ was set to 180 μs and the amplitude ratio α was set to 0.7.

**Figure 13:**
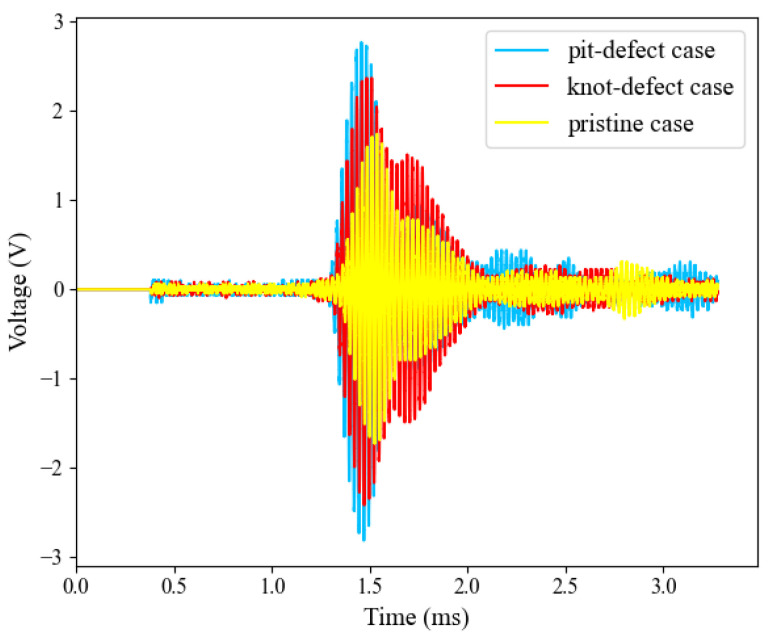
A-scan signals of pristine, knot-defect, and pit-defect cases.

After determining the excitation signal parameters, A-scan measurements were performed on pristine areas, knot defects, and pit defects of the wood surface. Figure 13 shows the response characteristics of the different regions to the ultrasonic signal. The results show that when the ultrasonic signal penetrated through the wood, the defect-free region exhibited a higher signal voltage, while the knot region had a significantly lower voltage response due to the complexity of the internal structure and higher attenuation characteristics. In addition, in the pit region, the signal voltage was higher than that in the defect-free region because the thickness of the board was thinner than that in the defect-free place. This suggests that different structural defects significantly affect the propagation characteristics of ultrasonic signals, and these differences provide critical data to support wood defect detection and classification.

### 4.3. C-Scan Detection of Wood with Composite Pulse Excitation

**Figure 14:**
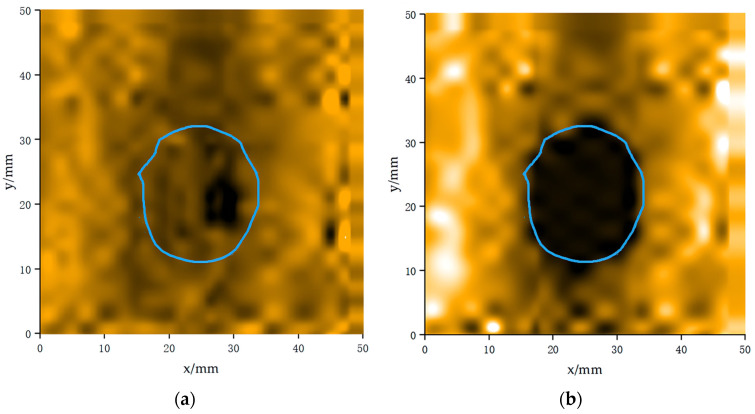
C-scan knot imaging maps of the transducer under different signal excitations: (**a**) Imaging results of knot defects with single-pulse signal excitation, and (**b**) imaging results of knot defects with double-pulse signal excitation.

In the experiments, a single-pulse signal and a composite signal were used as excitation signals for the ultrasound transducer to control the motor for C-scanning of the wood containing knot defects and the wood with pit defects. A data acquisition card was used to collect the excitation and received signals. The host computer analyzed the correlation between the excitation and received signals collected by the data acquisition card, calculated the maximum value of the correlation function, used the maximum value of the correlation result to generate the imaging data of the sampling points, obtained the imaging data of all the sampling points, and applied an interpolation algorithm to optimize the color imaging. In the node region of the wood, due to the significantly higher density of the node region compared to non-defect areas, the ultrasound wave experienced significant energy attenuation when passing through these areas. Therefore, when the ultrasonic wave passed through the part containing nodes, its voltage amplitude was lower than that of the defect-free region. In the ultrasonic imaging, this energy attenuation manifested as the node region appearing significantly darker, with the color tending toward black, thus creating a sharp contrast with the surrounding defect-free region, as shown in Figure 14b. The wireframe in the figure shows the actual size and location of the knot defect in the wood.

**Figure 15:**
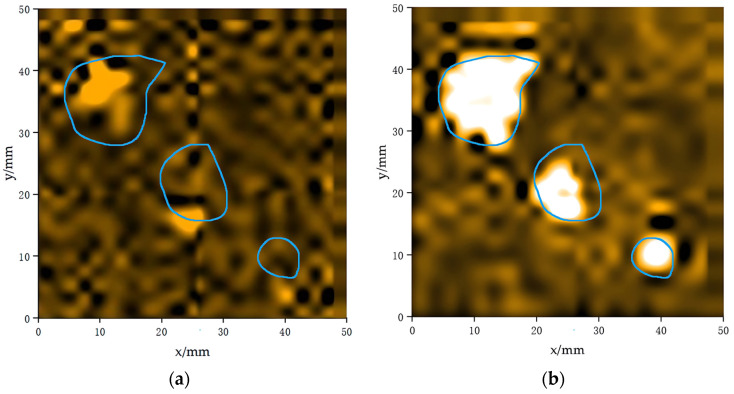
C-scan pit defect imaging maps of the transducer under different signal excitations: (**a**) Imaging results of pit defects with single-pulse signal excitation and (**b**) imaging results of pit defects with double-pulse signal excitation.

In the wood, the thickness at the pit areas was significantly thinner compared to that of the defect-free regions, which allowed the ultrasound waves to transmit more easily through these defective areas. When the ultrasound waves passed through these pits, the transmitted voltage amplitude appeared larger compared to the defect-free regions. In ultrasonic imaging, the pit areas appeared brighter, creating a sharp contrast with the surrounding defect-free regions, as shown in Figure 15b. The wireframe in the figure shows the actual size and location of the pit defect in the wood.

The results of the wood C-scan imaging system based on the composite pulse excitation technique agree with the theoretical expectations in the detection of knot and pit defects. The diameter of the knot defect measured by the system was about 24 mm, while the actual knot size was 21.8 mm. The relative accuracy of this measurement exceeds 90%. In addition, the system was capable of accurately detecting and reflecting the true shape of the pits and locating the position of pit defects. In contrast, when using single-pulse excitation, not all regions of the pit were fully covered or accurately measured, and the precision and accuracy of the knot imaging were reduced.

## 5. Conclusions and Future Work

In this study, the main controller generated a composite excitation pulse signal, and an air-coupled method was employed for nondestructive testing of wood. First, the principle of the composite pulse excitation technique was introduced, the output of the ultrasound transducer was mathematically analyzed, and the feasibility of the technique was verified through COMSOL simulations. The effects of the parameters *α* and $T_{d}$ on signal suppression were analyzed. Using wood samples with knot and pit defects as experimental objects, single-point tests were performed at knot, pit, and defect-free areas to determine the experimental parameters for wood with different defects. Finally, the differences in imaging results under two distinct excitation signals were verified through C-scan imaging of the wood plane. The experimental results showed that, compared to single-pulse excitation, the imaging accuracy of both defective and defect-free wood was significantly enhanced. In summary, the composite pulse excitation technique is an effective ultrasound signal-processing method that enhances the axial resolution of ultrasound waves and suppresses the impact of ring-down vibrations. This technique significantly improves the detection capabilities of the wood C-scan imaging system. Its advantages in enhancing inspection precision and accuracy provide a reliable means of inspection for the wood processing industry, thereby contributing to the quality and safety of wood products. The potential of this technique lies in its ability to effectively reduce the risk of missed detections and false positives of wood defects in actual production, optimizing the quality control process in wood processing.

## Figures and Tables

**Table 1:** Composite pulse parameters for ten preliminary test points for nodal defects.

Point	1	2	3	4	5	6	7	8	9	10
$T_{d}$/μs	132	156	132	156	132	180	132	156	180	132
α	0.8	0.6	0.6	0.7	0.8	0.6	0.7	0.7	0.8	0.8

**Table 2:** Composite pulse parameters for ten initial test points for pitting defects.

Point	1	2	3	4	5	6	7	8	9	10
$T_{d}$/μs	156	108	132	132	108	180	132	180	156	180
α	0.9	0.7	0.7	0.7	0.8	0.7	0.8	0.8	0.9	0.7

## Data Availability

Data are contained within the article.
